# Effects of recreational team sports on the metabolic health, body composition and physical fitness parameters of overweight and obese populations: A systematic review

**DOI:** 10.5114/biolsport.2024.134762

**Published:** 2024-02-12

**Authors:** Tao Wang, Liu Yang, Qi Xu, Jun Dou, Filipe Manuel Clemente

**Affiliations:** 1 Geely University of China, 641423 Chengdu, China; 2 Fuyang Normal University, 233600 Fuyang, China; 3 Gdansk University of Physical Education and Sport, 80-336 Gdańsk, Poland; 4 Nanchang Vocational University, 330000 Nanchang, China; 5 Sport Physical Activity and Health Research & Innovation Center, Viana do Castelo, Portugal; 6 Escola Superior Desporto e Lazer, Instituto Politécnico de Viana do Castelo, Rua Escola Industrial e Comercial de Nun’Álvares, 4900-347 Viana do Castelo, Portugal

**Keywords:** Group sport, Physical exercise, Cardiorespiratory fitness, Health, Overnutrition

## Abstract

This systematic review aims to provide a summary of the results from individual studies that specifically focused on overweight or obese populations, regardless of age or sex. The goal is to determine the effects of structured recreational team sports interventions (TSG) on metabolic health, body composition and physical fitness parameters when compared to passive or active control groups. This study adhered to the PRISMA guidelines for reporting a systematic review. A thorough examination of relevant literature was conducted on November 06, 2023, using three prominent databases: PubMed, Scopus, and the Web of Science. Inclusion criteria considered overweight (e.g., BMI 25.0–29.9 kg/m^2^) and obese (e.g., BMI > 30 kg/m^2^) populations exposed to training interventions using recreational team sports, while the comparator group consisted of the same populations not exposed to exercise (passive controls) or exposed to alternative training methods. The primary outcomes of interest were metabolic health parameters (glucose, waist circumference, blood pressure, cholesterol, triglycerides), body composition (e.g., fat mass, lean mass), as well as physical fitness parameters (e.g., aerobic fitness, muscular fitness). Only studies with two- or multi-arm designs, whether randomized or not, were eligible for inclusion. The PEDro scale was used to assess the methodological bias of the included studies. Out of the initial 275 titles retrieved, we deemed ten eligible for our study. In terms of body composition, TSG demonstrated a significant decrease in body mass index across three studies (−2.3 to −5.1%) and a significant reduction in waist circumference in four studies (−4.6% to −8.4%). Regarding blood pressure, TSG exhibited a significant decrease in systolic blood pressure in two studies (−3.9% to -8.3%), while diastolic blood pressure showed a significant decrease in only one study (−7.3%). Cholesterol levels saw a significant decrease in TSG in three studies (−7.0% to −9.7%), and triglyceride levels showed a significant reduction in four studies (−16.4% to −20.1%). In terms of aerobic fitness, TSG demonstrated within-group improvements in the field-based tests in three studies (8.1% to 79.0%), and within-group improvements in maximal oxygen uptake in four studies (6.5% to 31.0%), with significant favoring of TSG in most studies. Overall, TSG demonstrated significant benefits for overweight and obese populations compared to the control group, particularly in terms of improvements in body mass index, systolic blood pressures, cholesterol and triglyceride levels, and aerobic fitness. Future research ought to concentrate on tailoring responses to varying training volumes on an individualized basis.

## INTRODUCTION

Overweight and obesity represent a pervasive health concern in contemporary populations [1]. More than 1.9 billion adults aged 18 years and older are overweight, and of those, over 650 million adults are obese [2]. This prevalence represents a significant impact on both health [3] and physical fitness [4]. According to numerous scientific studies, the prevalence of overweight and obesity has risen steadily in recent decades, with an alarming increase in many parts of the world [5, 6].

While there are acknowledged limitations and concerns regarding the use of the Body Mass Index (BMI) as a surrogate for measuring body fat and determining overweight or obesity [7], it is worth noting that the Medical Subject Headings (MeSH) terms continue to define overweight as having a BMI in the range of 25.0–29.9 kg/ m^2^, with obesity being characterized by a BMI exceeding 30 kg/m^2^ (or 25% body fat percentage for men and 35% for women) [8].

The detrimental health effects of overweight and obesity are well-documented, and they encompass a wide range of conditions, including type 2 diabetes, cardiovascular disease, certain types of cancer, and musculoskeletal disorders [9, 10]. Physical exercise, when incorporated as part of comprehensive community programs, plays a pivotal role in mitigating the effects of overweight and obesity and its associated negative impact on health [11]. Scientific evidence underscores the effectiveness of physical exercise in combating overweight and obesity, not only by promoting weight loss but also by improving metabolic health and enhancing physical fitness [12].

Regular exercise contributes to energy expenditure [13], reduces body fat [14], and enhances insulin sensitivity [15], thereby addressing key components of the obesity problem. Furthermore, community-based programs, which offer structured and supportive environments for physical activity, hold the potential to bolster the impact of exercise interventions [16]. These programs often encompass educational components, social support networks, and opportunities for active recreation, fostering sustained adherence to physical activity [17]. In the fight against obesity, it is essential to recognize that physical exercise is just one facet of a multifaceted approach [18]. Non-pharmacological strategies for obesity management may also involve dietary modifications, behavioral therapy, and psychosocial support, as they can complement physical activity interventions to achieve more comprehensive and sustainable results [19]. Collaborative efforts within communities, incorporating various non-pharmacological approaches, are key to addressing the complex and multifactorial nature of obesity.

In the realm of physical exercise, scientific evidence supports the significant positive impact of aerobic exercise, particularly including intermittent forms and recreational sports, on metabolic health [20], physical fitness [21], and body composition [22] in overweight and obese populations. Numerous studies have shown that including intermittent forms of aerobic-based exercise and recreational sports has been shown to enhance insulin sensitivity [23], reduce fasting blood glucose levels [24], and improve lipid profiles [21], which are particularly relevant for individuals with overweight and obesity who often face heightened metabolic risks. Moreover, aerobic intermittent forms and recreational sports is highly effective in promoting physical fitness [25]. It has been associated with increased cardiorespiratory fitness [26] and muscle strength [27]. In terms of body composition, aerobic intermittent forms and recreational sports has demonstrated its ability to reduce body fat percentage while preserving lean muscle mass [21].

Participation in recreational team sports (e.g., soccer, basketball) presents a valuable strategy to combat sedentary behavior while offering numerous positive effects for such individuals, both physically and mentally [28]. Engaging in team sports not only provides an enjoyable and motivating alternative to traditional exercise but also fosters social interactions and a sense of community, which can be particularly beneficial for those dealing with isolation and self-esteem issues [29]. The engaging factors of team sports, such as competition, camaraderie, and the fun of the game, can enhance adherence to a physically active lifestyle among the obese and overweight.

In the context of a systematic review, summarizing the evidence regarding the effects of recreational team sports on overweight and obese individuals is essential and has been done [30]. Despite the abundance of systematic reviews and meta-analyses on physical activity and obesity [31, 32], there appears to be a notable gap: no review has exclusively focused on the benefits of recreational team sports for this specific population. Notably, several systematic reviews on recreational sports, such as soccer [33–36], have been disseminated, but without a targeted emphasis on a specific population or an assessment of its effects on particular outcomes.

Therefore, it is crucial to comprehend how metabolic health, encompassing variables such as waist circumference, fasting blood glucose, systolic and diastolic blood pressure, triglycerides, and highdensity lipoprotein cholesterol, along with considerations of body composition (e.g., fat mass, lean mass, fat-free mass) and physical fitness (e.g., cardiorespiratory fitness, muscular strength and resistance), may be influenced by SSGs programs implemented in overweight and obese populations. This research may potentially alleviate the adverse effects of obesity on functional and health parameters.

A dedicated systematic review in this domain could significantly advance the state of the art by providing a comprehensive and evidence-based overview of the unique contributions of recreational team sports in metabolic health, body composition and physical fitness, shedding light on a currently underrepresented but promising area of research. As a result, the systematic review seeks to consolidate the primary findings concerning the effects of experimental programs utilizing recreational sports like basketball and soccer, when compared to control groups, on the metabolic health, body composition, and physical fitness of overweight and obese individuals, irrespective of age or sex.

## MATERIALS AND METHODS

### Design and protocol registration

Adhering to the criteria established by the PRISMA 2020 guidelines [37, 38], widely acknowledged as the leading standard for transparent reporting in systematic reviews, we executed our systematic review. Furthermore, the present systematic review adheres to the recommended guidelines for reporting systematic reviews in sports sciences [39]. A comprehensive protocol governing our review procedures has been formally recorded on the Open Science Framework (OSF) platform, and it is identified with the project number osf. io/ack4t, accompanied by a DOI: 10.17605/OSF.IO/ACK4T.

### Eligibility criteria

In our systematic review, we have encompassed all original articles that have been published in peer-reviewed journals, even those in “ahead-of-print” status. We intentionally refrained from imposing any language restrictions to ensure the inclusion of a comprehensive range of articles.

Our approach to establishing eligibility criteria adhered to the PICOS framework, and it is possible to find a comprehensive breakdown of these criteria in Table 1.

**TABLE 1:** Eligibility criteria based on PICOS.

	Inclusion criteria	Exclusion criteria
Population	Individuals of all age groups and sex who meet specific criteria for overweight (with a BMI of 25.0–29.9 kg/m^2^, as defined in Medical Subject Headings terms) or obesity (with a BMI exceeding 30 kg/m^2^, as defined in Medical Subject Headings terms). This criterion (or one based on fat mass as > 35% [61] or other reasonable criteria as BMI > +2 standard deviations above ageand sex-specific WHO reference medians or ≥ 85^th^ percentile [62]) should be incorporated into the eligibility criteria of the study to ensure that participants who are non-overweight are not inadvertently mixed into the sample.	Individuals who are neither overweight nor obese or studies integrating mixed population that includes both overweight/obese individuals and those who are not overweight or obese. Studies that have presented BMI levels according to specific criteria but have not explicitly reported those criteria for participants fail to ensure the inclusion of other populations (i.e., nonoverweight individuals) within the sample range.

Intervention/exposure	Exclusively performing recreational team sports such as soccer or basketball, utilized as a form of exercise intervention for individuals who are overweight or obese.	Combined training programs, comprising recreational team sports and other training methods (e.g., running training, resistance training), or recreational team sports used alongside supplementary strategies like dietary modifications or medication.

Comparator	Comparators were considered for both cases: passive controls (a parallel group with similar characteristics not exposed to any exercise intervention) and/or active controls (a parallel group with similar characteristics exposed to a different form of exercise intervention unrelated to recreational team sports).	Combined training programs, comprising recreational team sports and other training methods (e.g., running training, resistance training), or other training interventions/control conditions in which a special dietary modification or medication was implemented.

Outcomes	We focused on parameters related to physical fitness (e.g., aerobic fitness, muscular fitness), metabolic health (glucose levels, waist circumference, blood pressure, cholesterol, triglycerides), and body composition (e.g., fat mass, lean mass), assessed at least in two time-points (baseline and post-intervention.	Immediate responses to a single session, or chronic adaptations encompassing outcomes beyond those delineated in the inclusion criteria (e.g., well-being and cognitive functioning).

Study design	The review will include studies that utilize either two-arm or multi-arm designs, irrespective of their randomization status.	Quasi-experimental studies, non-controlled studies, and observational studies will be excluded from consideration for inclusion.

We assessed the suitability of the articles using the PICOS approach, as delineated in Table 1. This systematic method facilitated a meticulous examination of the content, enabling us to establish well-defined inclusion and exclusion criteria. In order to ensure a comprehensive evaluation, we conducted a thorough review of the full texts to determine their eligibility for inclusion in the review.

### Information sources and search strategy

Our process for identifying relevant studies involved an extensive search conducted across several databases, including: (i) PubMed, (ii) Scopus, and (iii) Web of Science, up to the date of November 06, 2023. The searches were independently conducted by two authors, TW and FMC, on the same day. To augment the comprehensiveness of our methodology and minimize the potential of overlooking pertinent materials, we also conducted manual searches in the same day within the reference lists of the studies included in our review.

The search was systematically executed using Boolean operators AND/OR, with a deliberate decision to abstain from employing filters or constraints related to publication dates or language. This intentional approach was chosen to heighten the probability of discovering relevant studies. A comprehensive breakdown of the search strategy is meticulously documented in Table 2, conveniently presented below for reference.

**TABLE 2:** Full search strategy for each database.

Database	Specificities of the databases	Search Strategy	Number of articles
PubMed	Search for title and abstract also includes keywords	((“team sport*” [Title/Abstract] OR football* [Title/Abstract] OR soccer [Title/Abstract] OR futsal [Title/Abstract] OR handball* [Title/Abstract] OR volleyball* [Title/Abstract] OR basketball* [Title/Abstract] OR hockey [Title/Abstract] OR hurling [Title/Abstract] OR rugby [Title/Abstract] OR cricket [Title/Abstract] OR polo [Title/Abstract] OR lacrosse [Title/Abstract] OR softball [Title/Abstract] OR korfball [Title/Abstract] OR Gaelic* [Title/Abstract] OR netball [Title/Abstract] OR baseball [Title/Abstract] OR “sepak takraw” [Title/Abstract]) AND (Recreational [Title/Abstract] OR walk* [Title/ Abstract])) AND (Obes* [Title/Abstract] OR overweight [Title/Abstract])	51

Scopus	Search for title and abstract also includes keywords	( TITLE-ABS-KEY ( “team sport*” OR football* OR soccer OR futsal OR handball* OR volleyball* OR basketball* OR hockey OR hurling OR rugby OR cricket OR polo OR lacrosse OR softball OR korfball OR gaelic* OR netball OR baseball OR “sepak takraw” ) AND TITLE-ABS-KEY ( recreational OR walk* ) AND TITLE-ABSKEY ( obes* OR overweight ) )	117

Web of Science	Search for title and abstract also includes keywords and its designated “topic”	“team sport*” OR football* OR soccer OR futsal OR handball* OR volleyball* OR basketball* OR hockey OR hurling OR rugby OR cricket OR polo OR lacrosse OR softball OR korfball OR Gaelic* OR netball OR baseball OR “sepak takraw” (Topic) and Recreational OR walk* (Topic) and Obes* OR overweight (Topic)	107

### Selection process

The screening process was meticulously carried out by two assigned authors, specifically TW and LY. They independently assessed the retrieved records, encompassing both titles and abstracts. Following this, they individually evaluated the full texts of the selected records. In cases where discrepancies in the evaluation surfaced, a collaborative reevaluation process was initiated to reach a consensus. If a consensus could not be reached, the final decision was entrusted to a third author, FMC.

For efficient record management, we utilized EndNote X9.3.3 software, developed by Clarivate Analytics in Philadelphia, Pennsylvania, USA.

### Data collection process

The data collection process was conducted independently by the authors TW and LY. In instances where disagreements arose during this phase, FMC acted as a mediator to resolve any discrepancies. To enhance efficiency and maintain organization throughout this procedure, we utilized a dedicated Microsoft^®^ Excel datasheet. This datasheet encompassed all pertinent data and essential information, ensuring a structured and effective approach to data management.

### Data items

We carried out an exhaustive data collection process, capturing an extensive array of participant details and contextual factors. These variables encompassed essential information such as publication date, primary research objectives, sample size, country of origin, age distribution, sex composition, clinical conditions (e.g., diabetes, hypertension), study design, and the competitive level of the participants.

In terms of intervention-related details, we documented information related to the program’s duration, training frequency, adherence to the training regimen, and various aspects of the training dosage. This included specific information on the duration, repetitions, rest intervals, intensity, frequency, and training density.

Additionally, we maintained comprehensive records of the comparison groups, which included details about both active and control groups. This encompassed specifics regarding the type of exercises, exercise intensity, and volume. In the case of passive control, we reported whether participants maintained their daily habits or were subjected to alternative approaches (e.g., receiving only documentation for lifestyle changes).

Our main emphasis in gathering primary outcome measures centered around parameters associated with physical fitness (e.g., aerobic fitness, muscular fitness), metabolic health (glucose levels, waist circumference, blood pressure, cholesterol, triglycerides), and body composition (e.g., fat mass, lean mass). These measures were collected both before and after the intervention, with a minimum of two time points for intervention study.

### Study risk of bias assessment

To assess the risk of bias in the studies included in our review, we employed the Physiotherapy Evidence Database (PEDro) scale, a wellvalidated and reliable tool. This scale evaluates eleven specific criteria, with ten of them contributing to the overall score assigned to each article. Scores on the PEDro scale range from 0, representing the lowest quality, to 10, signifying the highest quality. Typically, predetermined score thresholds are utilized to categorize articles into qualitative categories, including ‘poor’ (< 4 points), ‘fair’ (4–5 points), ‘good’ (6–8 points), and ‘excellent’ (9–10 points).

The assessment process involved two authors independently evaluating and scoring the articles using the PEDro scale. Subsequently, these two authors (TW and LY) compared their individual scores and engaged in comprehensive discussions to resolve any discrepancies, criterion by criterion. In instances where a consensus could not be reached, a third author (FMC) was consulted to provide their score and ultimately make the definitive decision regarding the rating. The concordance between both authors (TW and LY) was assessed through the calculation of the kappa agreement rate between reviewers, yielding a k value of 0.96. Cohen’s kappa coefficient (k) is employed to gauge inter-rater reliability for qualitative (categorical) items. A k value approaching 1 indicates greater inter-rater variability.

### Data synthesis

Utilizing the data (mean ± standard deviation) obtained from preintervention (A) to post-intervention (B) within each group, we independently calculated the percentage of change for each individual study and outcome employing the following formula:

$$\left|\frac{B-A}{\frac{A+B}{2}}\right|\times 100$$

Furthermore, employing the data (mean ± standard deviation) derived from post-intervention for both the SSGs and control groups, we calculated Cohen’s d using the following formula [40]:

$$Cohen’s\;d=\frac{mean\;difference}{Pooled\;standard\;deviation}$$

## RESULTS

### Study Identification and Selection

**FIG. 1:**
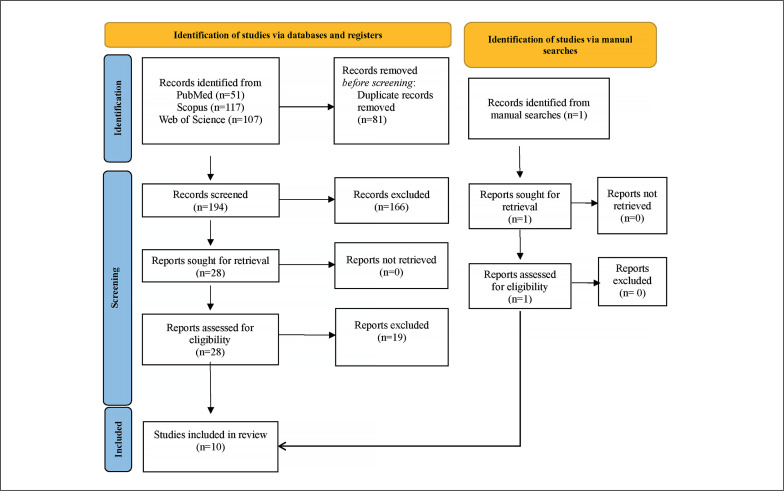
PRISMA flow diagram.

In Figure 1, we present the results of our initial search, which yielded a total of 275 titles. Following this initial step, we embarked on a thorough curation process, combining both automated and manual methods, in order to eliminate duplicate entries. This process resulted in the removal of 81 titles, leaving us with a collection of 194 unique titles. Each of these titles then underwent a meticulous assessment, considering both their titles and abstracts, to determine their relevance. Following this assessment, 166 studies were deemed unsuitable and subsequently excluded from our review.

The next stage of our investigation involved a comprehensive examination of the full texts of the remaining 28 studies. This systematic review ultimately identified 9 studies that met our predefined eligibility criteria. In addition to our database screening, we also conducted an extensive manual search within the references cited in the selected articles. This supplementary search uncovered one additional articles that satisfied our inclusion criteria. As a result, our systematic review incorporated a total of 10 articles. For a detailed breakdown of the full-text screening process and the rationale behind exclusions, we kindly refer you to Supplementary Material 1.

### Assessment of the risk of bias

In the course of our systematic review, we conducted an extensive examination of a set of ten studies, as detailed in Table 3. Within this collection, we identified that three studies exhibited a quality rating in the range of 4 to 5 points, signifying a moderate level of quality. Conversely, the remaining seven studies were assigned quality scores between 6 and 8 points, indicating a notably higher level of quality. It is worth emphasizing a shared limitation present in all the studies under scrutiny, which pertained to the absence of reported information concerning blinding procedures for both study participants and the individuals responsible for conducting the investigation and assessment.

**TABLE 3:** Physiotherapy Evidence Database (PEDro) scale ratings.

Study	C1	C2	C3	C4	C5	C6	C7	C8	C9	C10	C11	Score
Cvetkovic et al. [25]	1	1	1	0	0	0	0	1	1	1	1	6
Hansen et al. [41]	1	0	0	0	0	0	1	1	1	1	1	5
Hornstrup et al. [48]	1	1	0	0	0	0	1	1	1	1	1	6
Mohr et al. [49]	1	1	0	1	0	0	0	1	1	1	1	6
Moller et al. [47]	1	0	0	1	0	0	0	1	1	1	1	5
Seabra et al. [42]	1	0	0	0	0	0	0	1	1	1	1	4
Soares et al. [46]	1	1	0	1	0	0	0	1	1	1	1	6
Soares et al. [43]	1	1	0	1	0	0	0	1	1	1	1	6
Vasconcellos et al. [44]	1	1	1	1	0	0	1	1	1	1	1	8
Vasconcellos et al. [45]	1	1	1	1	0	0	1	1	1	1	1	8

C1: eligibility criteria were specified; C2: subjects were randomly allocated to groups; C3: allocation was concealed; C4: the groups were similar at baseline regarding the most important prognostic indicators; C5: there was blinding of all subjects; C6: there was blinding of all therapists who administered the therapy; C7: there was blinding of all assessors who measured at least one key outcome; C8: measures of at least one key outcome were obtained from more than 85% of the subjects initially allocated to groups; C9: all subjects for whom outcome measures were available received the treatment or control condition as allocated, or, where this was not the case, data for at least one key outcome were analyzed according to “intention to treat”; C10: the results of between-group statistical comparisons are reported for at least one key outcome; C11: the study provides both point measures and measures of variability for at least one key outcome. Score: The score is derived by summing the scores from C2 to C11, as recommended by the PEDro scale.

### Characterization of the individual studies

The table 4 summarizes the individual studies investigating the effects of team sports interventions on overweight and obese populations.

**TABLE 4:** Characteristics of the included studies

Study	N	Age (years old)	Sex	Team sport	Experimental groups (n)	Passive control groups (n)	Randomization	Study duration (w)	Outcomes extracted	Instruments/tests for measuring the outcomes
Cvetkovic et al. [25]	35	11–13	Male	Soccer	Soccer group (*n* = 10) HIIT group (*n* = 11)	Passive control (*n* = 14)	Yes	12	BMI (kg/m^2^) Body fat mass (%) Lean body mass () Muscle mass (%) CMJ arm swing (cm) Agility T-test (s) Sit and reach (cm) YYIRT (m) Systolic and diastolic blood pressure (mmHg) BMI (kg/m^2^)	Anthropometry (BMI) Bioelectrical impedance analyzer (for body fat mass, lean body mass, muscle mass) Agility t test Sit and reach test YYIRT Digital blood pressure monitor

Hansen et al. [41]	31	8–12	Both	Soccer	Soccer group (*n* = 20)	Passive control (*n* = 11)	No	12	BMI (kg/m^2^) Systolic and diastolic blood pressure (mmHg) BMI (kg/m^2^)	Anthropometry (BMI) Digital blood pressure monitor Anthropometry (BMI)

Hornstrup et al. [48]	32	41.8–46.3	Female	Handball	Handball experienced group (*n* = 13) Handball unexperienced group (*n* = 10)	Passive control (*n* = 9)	Yes	16	Systolic and diastolic blood pressure (mmHg) Gynoid fat mass (kg) BMI (kg/m^2^) Total cholesterol (mmol/L) Triglycerid (mmol/L) ${\dot{\text{V}}\text{O}}_{2\max}$ (ml/kg/min) YYIET (m)	X-ray absorptiometry (fat mass); Digital blood pressure monitor; Blood sample Incremental treadmill test (${\dot{\text{V}}\text{O}}_{2\max}$) YYIET

Mohr et al. [49]	41	35–50	Female	Soccer	Soccer group (*n* = 21)	Passive control (*n* = 20)	Yes	15	Systolic and diastolic blood pressure (mmHg) Total fat mass (kg) Lean body mass (kg) YYIET (m) Triglycerid (mmol/L) Total cholesterol (mmol/L)	Digital blood pressure monitor X-ray absorptiometry (fat and lean mass) YYIET Blood sample

Moller et al. [47]	68	58–61	Male	Multiple	Multiple sports group (*n* = 36) Aerobic training group (*n* = 32)	NA	No	12	Systolic and diastolic blood pressure (mmHg) Fat mass (kg) Lean body mass (kg) ${\dot{\text{V}}\text{O}}_{2\max}$ (ml/kg/min) 6-minute walking test (m) Balance (s) Sit-to-stand (stand/30 s)	Digital blood pressure monitor X-ray absorptiometry (fat and lean mass) Incremental cycling test (${\dot{\text{V}}\text{O}}_{2\max}$) 6-minute walking test Tandem balance test Sit-to-stand test

Seabra et al. [42]	88	8–12	Male	Soccer	Soccer group (*n* = 29) Traditional activity group (*n* = 29)	Passive control (*n* = 30)	No	24	BMI (kg/m^2^) Waist circumference (cm) Body fat (%) Lean mass (kg) Systolic and diastolic blood pressure (mmHg) Glucose (mg/dl) Triglycerid (mmol/L) Total cholesterol (mmol/L) ${\dot{\text{V}}\text{O}}_{2\max}$ (ml/kg/min)	Anthropometry (BMI; waist circumference) X-ray absorptiometry (fat and lean mass) Digital blood pressure monitor Blood sample Incremental treadmill test (${\dot{\text{V}}\text{O}}_{2\max}$)

Soares et al. [46]	39	60 ± 6	Both	Soccer	Soccer group* (*n* = 13) HIIT group* (*n* = 11)	Passive control* (*n* = 13)	Yes	12	BMI (kg/m^2^) Waist circumference (cm) Body fat (%) Lean mass (%) Glucose (mg/dl) Triglycerid (mmol/L) Total cholesterol (mmol/L)	Anthropometry (BMI; waist circumference) X-ray absorptiometry (fat and lean mass) Blood sample

Soares et al. [43]	18	12–17	Both	Soccer	Soccer group (*n* = 10)	Passive control (*n* = 8)	Yes	12	BMI (kg/m^2^) Waist circumference (cm) Fat mass (%) ${\dot{\text{V}}\text{O}}_{2\max}$ (ml/kg/min)	Anthropometry (BMI; waist circumference) X-ray absorptiometry (fat mass) Ramp-incremented protocol on a cycle ergometer (${\dot{\text{V}}\text{O}}_{2\max}$)

Vasconcellos et al. [44]	30	12–17	Both	Soccer	Soccer group (*n* = 10)	Passive control (*n* = 10)	Yes	12	BMI (kg/m^2^) Waist circumference (cm) Fat percentage (%) Systolic and diastolic blood pressure (mmHg) ${\dot{\text{V}}\text{O}}_{2\text{peak}}$ (ml/kg/min) Triglycerid (mmol/L) Total cholesterol (mmol/L) Fasting glucose (mg/dl)	Anthropometry (BMI; waist circumference) X-ray absorptiometry (fat percentage) Digital blood pressure monitor Blood sample Incremented protocol on a cycle ergometer (${\dot{\text{V}}\text{O}}_{2\max}$) Blood sample

Vasconcellos et al. [45]	13	13–17	Both	Soccer	Soccer group (*n* = 6)	Passive control (*n* = 7)	Yes	12	BMI (kg/m^2^) Waist circumference (cm) Waist circumference (cm) Systolic and diastolic blood pressure (mmHg) Triglycerid (mmol/L) Glucose (mg/dl)	Anthropometry (BMI; waist circumference) Digital blood pressure monitor Blood sample

BMI: body mass index; NA: not applicable; HIIT: high-intensity interval training;

* these groups had dietary plan; CMJ:countermovement jump; YYIRT: yo-yo intermittent recovery test; YYIET: Yo-Yo Intermittent endurance test; LDL: low-density lipoprotein; HDL: high-density lipoprotein

The age range across the studies varies, from 8 (Hansen et al. [41]) to 61 (Moller et al. [11]) years old, with the majority focusing on children and adolescents (Cvetkovic et al. [25]; Hansen et al. [41]; Seabra et al. [42]; Soares et al. [43]; Vasconcellos et al. [44]; Vasconcellos et al. [45]).

The number of participants ranges from 13 (Vasconcellos et al. [45]) to 88 (Seabra et al. [42]), with team sports like soccer and handball being common choices. The sex distribution is diverse, with some studies including both males and females (Hansen et al. [41]; Soares et al. [46]; Soares et al. [43]; Vasconcellos et al. [44]; Vasconcellos et al. [45]). The duration of interventions ranges from 12 to 24 weeks, with 12 weeks being the most common.

In the studies presented (Table 5), the total training sessions varied across experiments, with the highest number being 72 sessions in Seabra et al. [42] soccer training group and the minimum being 24 sessions in Cvetkovic et al. [25] and Moller et al. [47] soccer training groups. The training interventions in TSG were structured, involving warm-ups, technical exercises, small-sided games, and cool-downs. Notably, the control groups engaged in regular physical education classes or did not participate in specific training. The soccer training groups emphasized skill development, teamwork, and practical soccer play, utilizing various formats (e.g., 3 v 3 or 4 v 4) and pitch sizes.

**TABLE 5:** Characteristics of the experimental programs and the control groups

Study	Training frequency (n/w)	Total of training sessions (n)	Experimental groups (description of the training process)	Passive control groups (description)
Cvetkovic et al. [25]	2	24	Soccer training group: Frequency: Three times per week (Monday, Wednesday, and Friday); Duration: Each training session lasted approximately 60 minutes; Components: Included a 10-minute low-intensity warm-up, followed by 4 × 8-minute periods of play interspersed with 2 minutes of passive rest, and ended with a 10-minute cool-down; Activity: Participants engaged in five-to-seven-a-side matches on artificial grass with specific pitch area and aspect ratio; Warm-up: Included moderate-intensity jogging, static and dynamic stretching, and acceleration running; Rules: Matches were played without allocation of playing positions and followed the general rules of football. High-intensity interval training: Frequency: Three sets of high-intensity interval runs during each training session. Interval Distances: Individually adjusted based on each participant’s maximal aerobic speed (MAS) determined by the Half-Cooper test; Training Volume Progression: Increased over three phases (weeks 0–4, weeks 5–8, and weeks 9–12). Intensity: Work intervals were at 100% MAS, interspersed with equivalent passive recovery at 0% MAS; The distance for each participant was individually calculated to maintain the same training intensity; Structure: Training sessions involved a warm-up, high-intensity interval runs, and a cool-down, similar to the football training group.	Participants in the control group continued their regular physical education (PE) classes twice per week during the study period. These classes were conducted under the guidance of the PE teacher according to the curriculum.

Hansen et al. [41]	4	48	Soccer training group: training sessions were administered 4 days per week during the school day. Each session included a warm-up, technical football exercises, small-sided football games, and a cool-down period. Exercise Intensity: The football training was designed to maintain the heart rate above 80% of the maximum heart rate.	Did not engage in formal sports activities during the study period, except for the compulsory sport curriculum at school (2 sessions of 45–90 minutes per week).

Hornstrup et al. [48]	2–3	32–48	Handball training group: The team handball training sessions involved a structured regimen, commencing with a 12 to 15-minute warm-up, followed by four sets of 10-minute match play periods, including 4 v 4 and 3 v 3 formats, conducted on a 14-meter wide and 19-meter long team handball pitch. These play periods were separated by 3-minute breaks, and it’s noteworthy that no hard tackles were permitted during the training sessions. Additionally, the training balls were of a lightweight and soft material, emphasizing safety and skill development.	Not described

Mohr et al. [49]	2.3–3.5	40–52	Soccer training group: Small-sided football games, varying from 4 v 4 to 10 v 10, conducted in competitive settings, with 60-minutes per session. Supervision: A trained football coach oversaw all training sessions.	Did not engage in any specific training during the 15-week intervention period

Moller et al. [47]	2	24	Soccer training group: Training sessions included warm-ups, technical exercises, one-on-one (1 v 1) exercises, and small-sided games (up to six-a-side) with short intervals and breaks. The sessions also emphasized theories on physical exercise, healthy diet, and flexibility exercises.	Did not engage in any specific training

Seabra et al. [42]	3	72	Soccer training group: The soccer training program consisted of three sessions per week for a duration of 60–90 minutes each. The structure consisted into warm-Up (10–20 minutes): Each session began with a warm-up to prepare participants for physical activity; Technical Drills and Small-Sided Games (40–60 minutes): The core of the soccer training involved technical drills and small-sided games. This part of the session focused on skill development, teamwork, and practical soccer plan; cool-Down (10 minutes): At the end of each session, a cool-down period was implemented to gradually reduce heart rate and prevent injury. The training intensity for the soccer program was designed to maintain heart rates at approximately 70–80% of the maximum heart rate, as confirmed through monitoring. Traditional activity program: The traditional activity program for the AS group involved three sessions per week, with each session lasting 60–90 minutes. The structure of the AS group sessions was as follows: warm-Up (10–20 minutes): Each session started with a warm-up period to prepare participants for physical activity; generalized activities (40–60 minutes): The core of the session focused on general activities aimed at improving various aspects of fitness, including aerobic endurance, coordination, balance, flexibility, and strength. These activities included walking, running, gymnastics, games, and others; cool-Down (10 minutes): At the conclusion of each session, there was a cool-down period to gradually lower heart rates and reduce the risk of injury. Similar to the soccer training group, the training intensity for the traditional group was designed to maintain heart rates at approximately 70–80% of the maximum heart rate, as monitored throughout the sessions.	Not applicable

Soares et al. [46]	3	36	Soccer training group: These sessions were characterized by moderate-tohigh intensity, lasting 40 minutes each, with a frequency of 3 sessions per week. During the training, participants maintained their heart rate above 60% of their maximum heart rate for approximately 70% of the total training time. The training sessions primarily involved ordinary small-sided friendly games (ranging from 3 v 3 to 7 v 7) and were conducted either outdoors on a natural grass pitch (30–40 meters wide and 45–60 meters long) or indoors on a wooden court in case of rainy weather. Notably, there were no goalkeepers involved in these sessions. Each training session followed a structured format, including a 10-minute low-intensity warm-up, followed by 3 minutes of passive rest, and then two 12-minute periods of play, with 3-minute passive rest intervals in between. High-intensity interval training: Each session initiated with a 10-minute warm-up designed to prepare participants for physical activity. Following the warm-up, the training sessions were divided into two distinct segments. First, participants engaged in a 12-minute session of high-intensity interval exercise, likely involving short bursts of intense physical activity interspersed with brief rest or lower-intensity intervals. Subsequently, the program transitioned into a 12-minute segment of continuous training performed at a moderate intensity level, similar to the average intensity of the soccer group. Before the commencement of each running session, all subjects participated in a 10-minute dynamic warm-up, which included activities like walking and running, progressively increasing in distance from 50 to 100 meters on a court. This comprehensive protocol aimed to deliver a structured and effective running training program.	Not described

Soares et al. [43]	3	36	Soccer training group: The exercise sessions had a duration of 60 minutes and followed a specific structure. They commenced with a 10-minute warm-up to prepare participants for physical activity. The core of each session consisted of 40 minutes of small-sided games, with variations like 2 vs. 2, 3 vs. 3, and 4 vs. 4. These small-sided games were played in confined pitch areas. The sessions concluded with a 10-minute cool-down period.	Not described

Vasconcellos et al. [44]	3	36	Soccer training group: Every session was structured with a 10-minute warm-up at the outset, followed by 40 minutes of engaging games conducted in compact pitch areas, including formats like 2 vs. 2, 3 vs. 3, and 4 vs. 4. The sessions then concluded with a 10-minute cool-down period.	Not described

Vasconcellos et al. [45]	3	36	Soccer training group: Each exercise session was structured to include a 10-minute warm-up, succeeded by 40 minutes of participation in small-sided games, featuring formats like 2 vs. 2, 3 vs. 3, and 4 vs. 4. These games were strategically played in smaller pitch areas, with the recognized benefits of enhancing aerobic fitness and honing technical and tactical skills. Notably, the sessions maintained an average percentage of maximal heart rate at 84.5 ± 4.1%, indicating a consistently high level of cardiovascular intensity throughout the games.	Not described

### Summary of the individual studies

In analyzing the statistical tendencies of using team sports groups (TSG) versus control groups (CG) across multiple studies (Table 6), several patterns emerge in regards body composition and anthropometrics. Looking at body mass index (BMI), Cvetkovic et al. [25], Soares et al. [43] and Vasconcellos et al. [44] show a significant decrease in BMI favoring TSG. Hansen et al. [41], Hornstrup et al. [48], and Seabra et al. [42] display no significant changes within TSG or CG or between groups. Soares et al. [46] demonstrate a decrease in BMI within TSG, although not significant from CG.

**TABLE 6:** Results of individual studies on anthropometry and body composition

Study	Variable	TSG pre	TSG post	%dif (post-pre)	Within-TSG (p-value)	CG pre	CG post	%dif (post-pre)	Within-CG (p-value)	Between groups (p-value)	ES between groups (post)
Cvetkovic et al. [25]	BMI (kg/m^2^)	25.43 ± 4.05	24.65 ± 4.22	−3.1%ß	p > 0.05	25.29 ± 4.77	26.16 ± 4.49	3.4%Ý	p > 0.05	p < 0.05 favoring TSG	−0.356

Hansen et al. [41]	BMI (kg/m^2^)	23.1 ±2.9	22.9 ±2.8	−0.9%ß	p > 0.05	28.0 ±2.9	27.6 ±3.3	−1.4%Ý	p > 0.05	p > 0.05	−1.320

Hornstrup et al. [48]#	BMI (kg/m^2^)	28.4 ±4.7	28.4 ±4.5	0.0%Þ	ND	29.8 ±5.5	30.3 ±5.7	1.7%Ý	ND	p > 0.05	−0.603

Seabra et al. [42]	BMI (kg/m^2^)	23.7 ±2.8	23.5 ±2.6	−0.8%ß	p > 0.05	25.1 ±3.8	25.3 ±4.3	0.8%ß	p > 0.05	p > 0.05	−0.743

Soares et al. [46]	BMI (kg/m^2^)	33.1 ±1.0	31.4 ±0.8	−5.1%ß*	*p < 0.05	33.3 ±1.2	31.4 ±1.1	−5.7%ß	*p < 0.05	p > 0.05	< 0.001

Soares et al. [43]	BMI (kg/m^2^)	30.7 ±5.0	29.4 ±4.8	−4.2%ß*	*p < 0.001	32.5 ±4.1	34.1 ±4.7	4.9%Ý*	*p < 0.001	p = 0.05 favoring TSG	−1.101

Vasconcellos et al. [44]	BMI (kg/m^2^)	31.1 ±5.2	30.4 ±4.2	−2.3%ß*	p = 0.024	32.2 ±4.9	33.5 ±5.8	4.0%Ý*	*p = 0.001	p < 0.001 favoring TSG	−0.884

Vasconcellos et al. [45]	BMI (kg/m^2^)	30.5 ±2.1	30.4 ±4.2	−0.3%ß	p > 0.05	30.8 ±3.1	32.5 ±5.8	5.5%Ý	p > 0.05	p > 0.05	−0.784

Cvetkovic et al. [25]	Body fat mass (%)	36.25 ± 6.70	33.47 ± 6.99	−7.6%ß	p > 0.05	29.94 ± 8.41	29.60 ± 7.68	−1.1%ß	p > 0.05	p > 0.05	0.527

Hornstrup et al. [48]#	Gynoid fat mass (kg)	5.9 ±1.8	5.8 ±1.8	−1.7%ß	ND	6.6 ±1.7	6.7 ±1.7	1.5%Ý	ND	p > 0.05	−0.529

Mohr et al. [49]	Total fat mass (kg)	36.6 ±2.0	35.7 ± ND	−2.7%ß*	*p < 0.05	33.0 ±1.6	33.2 ± ND	0.6%Ý	p > 0.05	p < 0.05 favoring TSG	

Moller et al. [47]$	Fat mass (kg)	36.8± 14.5	35.9 ± ND	−2.4%ß*	*p < 0.05	39.1 ± 16.2	37.5 ± ND	−4.1%ß*	*p < 0.05	p > 0.05	

Seabra et al. [42]	Body fat (%)	34.3 ± 5.6	32.1 ± 5.7	−6.4%ß*	*p < 0.05	34.4 ± 7.6	37.5 ± 10.5	9.0%Ý*	*p < 0.05	*p < 0.05 favoring TSG	−0.639

Soares et al. [46]	Body fat (%)	34.3 ± 1.7	31.8 ± 2.1	−7.3%ß*	*p < 0.05	36.2 ± 2.0	34.3 ± 1.9	−5.3%ß	*p < 0.05	p > 0.05	−1.072

Soares et al. [43]	Body fat (%)	41.4 ± 5.8	39.2 ± 5.5	−5.3%ß*	*p = 0.001	42.1 ± 5.5	41.9 ± 5.2	−0.5%ß	p > 0.05	p > 0.05	−0.427

Vasconcellos et al. [44]	Fat (%)	41.1 ± 6.1	38.9 ± 6.1	−5.3%ß*	*p < 0.001	42.0 ± 5.4	41.7 ± 5.1	−0.7%ß	p > 0.05	p = 0.024 favoring TSG	−0.483

Vasconcellos et al. [45]	Fat (%)	42.3 ± 7.2	38.9 ± 6.1	−8.0%ß	p > 0.05	40.9 ± 7.2	41.7 ± 5.1	1.9%Ý	p > 0.05	p > 0.05	−0.483

Seabra et al. [42]	Waist circumference (cm)	83.6 ± 9.4	79.4 ± 9.5	−5.0%ß*	*p < 0.05	85.7 ± 11.7	85.5 ± 12.3	−0.2%ß	p > 0.05	p < 0.05 favoring TSG	−0.554

Soares et al. [46]	Waist circumference (cm)	109.0 ± 3.1	104.0 ± 2.6	−4.6%ß*	*p < 0.05	106.5 ± 2.3	101.2 ± 2.0	−5.0%ß	*p < 0.05	p > 0.05	1.182

Soares et al. [43]	Waist circumference (cm)	97.9 ± 9.8	91.4 ± 9.9	−6.7%ß*	p = 0.003	106.6 ± 13.2	106.0 ± 13.6	−0.6%ß	p > 0.05	p = 0.02 favoring TSG	−1.116

Vasconcellos et al. [44]	Waist circumference (cm)	98.7 ± 10	90.5 ± 8.9	−8.4%ß*	p = 0.004	103.5 ± 14.5	101.9 ± 17.0	−1.5%ß	p > 0.05	p > 0.05	−1.147

Vasconcellos et al. [45]	Waist circumference (cm)	99.2 ± 9.6	87.5 ± 10.9	−11.8%ß	p > 0.05	101.5 ± 12.3	107.9 ± 17.0	6.3%Ý	p > 0.05	p > 0.05	−1.327

Cvetkovic et al. [25]	Lean body mass (%)	63.76 ± 6.72	66.50 ± 6.99	4.3%Ý	p > 0.05	69.96 ± 8.40	70.33 ± 7.67	0.5%Ý	p > 0.05	p > 0.05	−0.522

Cvetkovic et al. [25]	Muscle mass (%)	34.51 ± 3.70	36.03 ± 3.79	4.4%Ý	p > 0.05	38.42 ± 4.94	38.85 ± 4.58	1.1%Ý	p > 0.05	p > 0.05	−0.672

Mohr et al. [49]	Lean body mass (kg)	42.7 ± 1.4	43.3 ± ND	1.4%Ý	p > 0.05	43.7 ± 1.2	43.9 ± ND	0.5%Ý	p > 0.05	p > 0.05	N/A

Moller et al. [47]$	Lean body mass (kg)	58.5 ± 10.0	58.3 ± ND	−0.3%ß	p > 0.05	61.7 ± 12.4	61.3 ± ND	−0.6%ß	p > 0.05	p > 0.05	N/A

Seabra et al. [42]	Lean mass (%)	17.9 ± 4.7	19.4 ± 4.6	8.4%Ý	p > 0.05	18.4 ± 5.3	19.4 ± 5.1	5.4%Ý*	*p < 0.05	p > 0.05	< 0.001

Soares et al. [46]	Lean mass (kg)	59.6 ± 2.4	59.7 ± 2.3	0.2%Ý	p > 0.05	54.4 ± 3.0	53.3 ± 2.6	−2.0%ß	p > 0.05	p > 0.05	2.612

Vasconcellos et al. [44]	Fat free mass (kg)	43.2 ± 8.2	45.0 ± 8.0	4.2%Ý	p > 0.05	48.3 ± 7.5	49.9 ± 8.9	3.3%Ý	p > 0.05	p > 0.05	−0.578

TSG: team sports group; CG: control group; #unexperienced group; $: the control group was active and was enrolled at fitness classes; ND: not described; N/A: not applicable; ES: calculation of Cohen’s d for the post-intervention comparison between TSG and control groups.

Examining body fat metrics, several studies reveal a consistent pattern favoring TSG. Mohr et al. [49], Seabra et al. [42] and Vasconcellos et al. [44] report a decrease in body fat percentage favoring TSG. Cvetkovic et al. [25], Hornstrup et al. [48] and Vasconcellos et al. [45] reports no within-group or between group significant differences. Moller et al. [11, Soares et al. [46] and Soares et al. [43] show a significant improvement in TSG although not significantly different from CG. In terms of lean body mass and muscle mass, none of the studies presents significant differences in within-group analysis or between-groups analysis.

Waist circumference, another key measure, sees consistent favorable changes in TSG. Seabra et al. [42], and Soares et al. [43] report a significant decrease in waist circumference favoring TSG in regards between group differences. Soares et al. [46] and Vasconcellos et al. [44] presents significant improvements on TSG, although not significantly different from CG.

Looking at the results from various studies comparing the impact of TSG to CG on blood pressure, several trends emerge (Table 7). In terms of systolic blood pressure (SBP), the within-group differences in TSG varied across studies, with some showing a significant decrease as the cases of Mohr et al. [49] and Vasconcellos et al. [44], while others did not. Between-group comparisons indicated a significant favoring of TSG in terms of SBP in some studies as Hansen et al. [41], Mohr et al. [49], Moller et al. [47] and Vasconcellos et al. [44], highlighting the potential positive impact of team sports on SBP.

**TABLE 7:** Results of individual studies on blood pressure

Study	Variable	TSG pre	TSG post	%dif (post-pre)	Within-TSG (p-value)	CG pre	CG post	%dif (post-pre)	Within-CG (p-value)	Between groups (p-value)	ES between groups (post)
Cvetkovic et al. [25]	SBP (mm/Hg)	121.00 ± 11.74	117.50 ± 11.12	−2.9%ß	p > 0.05	123.93 ± 15.34	124.85 ± 11.58	0.7%Ý	p > 0.05	p > 0.05	−0.576

Hansen et al. [41]	SBP (mm/Hg)	114 ± 7	112 ± 9	−1.8%ß	p > 0.05	112 ± 6	122 ± 10	8.9%Ý*	*p = 0.02	p < 0.05 favoring TSG	−0.696

Hornstrup et al. [48]#	SBP (mm/Hg)	107 ± 14	110 ± 14	2.8%Ý	p > 0.05	112 ± 13	116 ± 14	3.6%Ý	p > 0.05	p > 0.05	−0.429

Mohr et al. [49]	SBP (mm/Hg)	139 ± 2	127.4 ±3.4	−8.3%ß*	*p < 0.05	134 ± 4	133.6 ±2.9	−0.3%ß	p > 0.05	p < 0.05 favoring TSG	−0.492

Moller et al. [47]$	SBP (mm/Hg)	129 ± 15	126.2 ± ND	−2.2%ß	p > 0.05	133 ± 13	135.8 ± ND	2.1%Ý	p > 0.05	p < 0.05 favoring TSG	N/A

Seabra et al. [42]	SBP (mm/Hg)	111.0 ± 12.4	111.4 ± 10.6	0.4Ý	p > 0.05	109.1 ± 15.3	110.5 ±8.9	1.3%Ý	p > 0.05	p > 0.05	0.056

Vasconcellos et al. [44]	SBP (mm/Hg)	128 ± 10	123 ± 13	−3.9%ß*	*p = 0.006	128 ± 9	129 ± 6	0.8%Ý	p > 0.05	p = 0.025 favoring TSG	−0.526

Vasconcellos et al. [45]	SBP (mm/Hg)	135 ± 4	128 ± 3	−5.2ß	p > 0.05	137 ± 6	137 ± 3	0.0%Þ	p > 0.05	p > 0.05	−0.690

Cvetkovic et al. [25]	DBP (mm/Hg)	70.00 ± 10.80	64.00 ± 3.94	−8.6%ß	p > 0.05	62.68 ± 9.29	64.57 ± 8.42	3.0%Ý	p > 0.05	p > 0.05	−0.148

Hansen et al. [41]	DBP (mm/Hg)	62 ± 8	63 ± 13	1.6%Ý	p > 0.05	67 ± 6	64 ± 7	−4.5%ß	p > 0.05	p > 0.05	−0.092

Hornstrup et al. [48]#	DBP (mm/Hg)	72 ± 9	70 ± 8	−2.8%ß	p > 0.05	79 ± 10	79 ± 11	1.3%Ý	p > 0.05	p > 0.05	−1.093

Mohr et al. [49]	DBP (mm/Hg)	86 ± 2	79.9 ±2.5	−7.3%ß*	*p < 0.05	82 ± 3	82.5 ±2.1	0.6%Ý	p > 0.05	p < 0.05 favoring TSG	−0.715

Moller et al. [47]$	DBP (mm/Hg)	78 ± 8	75.4 ± ND	−3.3%ß	p > 0.05	79 ± 8	79.2 ± ND	0.3%Ý	p > 0.05	p > 0.05	N/A

Seabra et al. [42]	DBP (mm/Hg)	57.6 ±8.6	53.7 ±7.5	−6.8%	p > 0.05	54.9 ± 10.3	56.7 ±6.2	3.3%Ý	p > 0.05	p > 0.05	-0.483

Vasconcellos et al. [44]	DBP (mm/Hg)	81 ± 5	79 ± 14	−2.5%ß	p > 0.05	79 ± 5	79 ± 4	-1.3%ß	p > 0.05	p > 0.05	< 0.001

Vasconcellos et al. [45]	DBP (mm/Hg)	87 ± 2	84 ± 3	−3.4%ß	p > 0.05	89 ± 4	88 ± 4	1.1%ß	p > 0.05	p > 0.05	-0.557

TSG: team sports group; CG: control group; #unexperienced group; $: the control group was active and was enrolled at fitness classes; ND: not described; N/A: not applicable; ES: calculation of Cohen’s d for the post-intervention comparison between TSG and control groups.

For diastolic blood pressure (DBP), within-group differences in TSG also varied, with one study showing a significant decrease Mohr et al. [49], while others did not. The between-group comparisons indicated a significant favoring of TSG in terms of DBP in one study Mohr et al. [49], suggesting that the majority of the studies does not confirm a significant and positive impact of team sports on DBP.

In terms of total cholesterol levels, within-group differences in TSG showed mixed results (Table 8), with some studies indicating a significant decrease as Seabra et al. [42], Soares et al. [46] and Vasconcellos et al. [44], while others did not as Hornstrup et al. [48] and Mohr et al. [49]. Between-group comparisons indicated a significant favoring of TSG in terms of total cholesterol in most of studies as Mohr et al. [49], Seabra et al. [42], Soares et al. [46] and Vasconcellos et al. [44], suggesting a potential positive impact of team sports on cholesterol levels.

**TABLE 8:** Results of individual studies on health biomarkers

Study	Variable	TSG pre	TSG post	%dif (post-pre)	Within-TSG (p-value)	CG pre	CG post	%dif (post-pre)	Within-CG (p-value)	Between groups (p-value)	ES between groups (post)
Hornstrup et al. [48]#	Total cholesterol (mmol/L)	5.3 ±0.8	5.6 ±0.9	5.7%Ý	ND	4.9 ±0.7	5.2 ±0.7	6.1%Ý	ND	p > 0.05	0.590

Mohr et al. [49]	Total cholesterol (mmol/L)	5.8 ±0.1	ND	N/A	ND	5.3 ±0.2	5.3 ±0.2	0.0%Þ	ND	p < 0.05 favoring TSG	N/A

Seabra et al. [42]	Total cholesterol (mg/dl)	171.6 ± 42.7	159.6 ± 32.1	−7.0%ß*	*p < 0.05	166.4 ± 21.3	173.1 ± 25.3	4.0%Ý	p > 0.05	p < 0.05 favoring TSG	−0.420

Soares et al. [46]	Cholesterol (mg/dl)	179.6 ±9.0	163.3 ±9.5	−9.1%ß*	*p < 0.05	181.7 ± 13.3	203 ± 14.6	11.8%Ý	p > 0.05	p < 0.05 favoring TSG	−1.150

Vasconcellos et al. [44]	Total cholesterol	166.4 ± 21.6	150.2 ± 25.2	−9.7%ß*	*p = 0.008	168.7 ± 32.7	181.4 ± 30.2	7.5%Ý*	*p = 0.021	p = 0.001 favoring TSG	−0.708

Hornstrup et al. [48]#	Triglycerid (mmol/L)	1.0 ±0.3	1.0 ±0.3	0.0%Þ	ND	1.0 ±0.5	1.1 ±0.7	10.0%Ý	ND	p > 0.05	−0.095

Mohr et al. [49]	Triglycerid (mmol/L)	1.3 ±0.1	ND	N/A	ND	1.0 ±0.1	1.0 ±0.1	0.0%Þ	ND	p < 0.05 favoring TSG	N/A

Seabra et al. [42]	Triglycerides (mg/dl)	70.9 ± 31.8	54.9 ± 15.9	−22.4%ß*	*p < 0.05	61.8 ± 26.5	74.0 ± 44.6	19.8%Ý	p > 0.05	p > 0.05	−0.573

Soares et al. [46]	Triglycerides (mg/dl)	140 ± 10.1	117.1 ± 13.9	−16.4%ß*	*p < 0.05	150.3 ± 23.0	164.6 ± 20.8	9.6%Ý	p > 0.05	p > 0.05	−0.783

Vasconcellos et al. [44]	Triglycerides (mg/dl)	118.4 ± 38.8	97.9 ± 19.6	−17.4%ß*	*p = 0.018	120.3 ± 72.6	145.4 ± 91.4	20.9%Ý*	*p = 0.008	p = 0.001 favoring TSG	−0.695

Vasconcellos et al. [45]	Triglycerides (mg/dl)	173.0 ± 22.1	138.3 ± 19.3	−20.1%ß*	*p = 0.02	189.1 ± 31.7	185.4 ± 21.4	−1.9%ß	p > 0.05	p = 0.05 favoring TSG	−0.839

Seabra et al. [42]	Glucose (mg/dl)	80.6 ±5.5	83.4 ±8.1	3.5%Ý	p > 0.05	82.6 ±7.0	82.5 ±5.3	−0.1%ß	p > 0.05	p > 0.05	0.011

Soares et al. [46]	Glucose plasma (mg/dl)	167 ±3.0	135 ±7.5	−19.2%ß*	*p < 0.05	172 ±9.9	141 ±4.3	−17.8%ß*	*p < 0.05	p > 0.05	−0.155

Vasconcellos et al. [44]	Fasting glucose (mg/dl)	92.9 ±6.4	91.9 ±6.4	−1.1%ß	p > 0.05	87.6 ±8.9	94.3 ±8.3	7.7%Ý*	p = 0.007	p = 0.029 favoring TSG	−0.235

TSG: team sports group; CG: control group; #unexperienced group; ND: not described; N/A: not applicable; ES: calculation of Cohen’s d for the post-intervention comparison between TSG and control groups.

For triglyceride levels, within-group differences in TSG showed a significant decrease across studies as Mohr et al. [49], Vasconcellos et al. [44] and Vasconcellos et al. [45]. Between-group comparisons indicated a significant favoring of TSG in terms of triglyceride levels in Mohr et al. [49], Vasconcellos et al. [44] and Vasconcellos et al. [45], highlighting a potential positive impact of team sports on triglyceride levels.

In terms of glucose levels, within-group differences in TSG varied, with one study showing a significant decrease as Vasconcellos et al. [44] and others not as Seabra et al. [42] and Soares et al. [46]. Between-group comparisons indicated a significant favoring of TSG in terms of fasting glucose levels Vasconcellos et al. [44].

In terms of aerobic fitness (Table 9), the Yo-Yo Intermittent Endurance Test (YYIET) and the Yo-Yo Intermittent Recovery test (YYIRT) showed within-group improvements in TSG as demonstrated in Hornstrup et al. [6], Cvetkovic et al. [25] and Mohr et al. [49], with significant increases in distance covered and being consistently favored to TSG, indicating a significant positive impact on aerobic fitness in comparison to CG.

**TABLE 9:** Results of individual studies on physical fitness

Study	Variable	TSG pre	TSG post	%dif (post-pre)	Within-TSG (p-value)	CG pre	CG post	%dif (post-pre)	Within-CG (p-value)	Between groups (p-value)	ES between groups (post)
Cvetkovic et al. [25]	YYIRT (m)	476.0 ± 182.4	856.0 ± 456.0	79.0%Ý*	*p = 0.025	722.8 ± 576.0	857.2 ± 542.0	18.6%Ý	p > 0.05	p < 0.005 favoring TSG	−0.004

Hornstrup et al. [48]#	YYIET (m)	650 ± 342	890 ± 454	36.9%Ý*	*p < 0.01	474.7 ± 237.4	479.1 ± 219.8	0.9%Ý	ND	p < 0.005 favoring TSG	1.307

Mohr et al. [49]	YYIET (m)	420 ± 45	454 ± 81	8.1%Ý*	*p < 0.05	458 ± 43	453 ± 42	−1.1%ß	p > 0.05	p < 0.005 favoring TSG	0.008

Hornstrup et al. [48]#	${\dot{\text{V}}\text{O}}_{2\max}$ (ml/kg/min)	29.4 ±5.8	31.3 ±5.0	6.5%Ý*	*p < 0.01	29.1 ±5.0	28.5 ±4.7	−2.1%ß	ND	p < 0.005 favoring TSG	0.144

Moller et al. [47]$	${\dot{\text{V}}\text{O}}_{2\max}$ (ml/kg/min)	21.7 ±5.4	22.2 ± ND	2.3%Ý	p > 0.05	22.4 ±5.9	23.5 ± ND	4.9%Ý	p > 0.05	p > 0.05	N/A

Seabra et al. [42]	${\dot{\text{V}}\text{O}}_{2\max}$ (ml/kg/min)	44.7 ±8.5	50.4 ±5.6	12.7%Ý*	*p < 0.05	48.6 ± 11.5	50.7 ± 13.9	4.3%Ý	p > 0.05	p > 0.05	−0.053

Soares et al. [46]	${\dot{\text{V}}\text{O}}_{2\max}$ (ml/kg/min)	24.6 ±3.5	30.4 ±5.7	23.6%Ý*	*p < 0.001	23.3 ±3.4	24.9 ±4.1	6.9%Ý	p > 0.05	p = 0.013 favoring TSG	1.042

Vasconcellos et al. [44]	${\dot{\text{V}}\text{O}}_{2\text{peak}}$ (ml/kg/min)	25.2 ±3.2	33.1 ±9.2	31.0%Ý*	*p < 0.001	22.9 ±3.1	24.0 ±3.9	4.8%Ý	p > 0.05	p = 0.013 favoring TSG	1.334

Cvetkovic et al. [25]	CMJ arm swing (cm)	17.57 ± 4.24	20.56 ± 3.62	17.0%Ý	p > 0.05	21.42 ± 6.72	24.86 ± 5.68	15.9%Ý	p > 0.05	p > 0.05	−0.728

Cvetkovic et al. [25]	Agility T-test (s)	8.58 ± 1.05	7.67 ± 0.63	−10.6%ß*	*p = 0.031	8.06 ± 0.57	7.66 ± 0.49	−5.0%ß	p > 0.05	p < 0.005 favoring TSG	0.002

Cvetkovic et al. [25]	Sit and reach (cm)	5.00 ± 3.73	8.93 ± 6.27	78.6%Ý	p > 0.05	10.33 ± 6.97	12.51 ± 7.18	21.1%Ý	p > 0.05	p > 0.05	−0.698

Moller et al. [47]$	STS (stand/30 s)	ND	+1.5	N/AÝ	p > 0.05	ND	+3.5	N/A Ý*	*p < 0.05	p < 0.005 favoring CG$	N/A

Moller et al. [47]$	Balance (s)	ND	+3.6	N/A Ý*	*p < 0.05	ND	+4.5	N/A Ý*	*p < 0.05	p > 0.05	N/A

Moller et al. [47]$	6MWT (m)	ND	+27.2	N/A Ý*	*p < 0.05	ND	+54.4	N/A Ý*	*p < 0.05	p > 0.05	N/A

TSG: team sports group; CG: control group; YYIRT: Yo-Yo intermittent recovery test; YYIET: Yo-Yo Intermittent endurance test; ${\dot{\text{V}}\text{O}}_{2\max}$: maximal oxygen uptake; #unexperienced group; STS: sit-to-stand. 6MWT: 6-minute walking test; $: the control group was active and was enrolled at fitness classes; #unexperienced group; N/A: not applicable; ES: calculation of Cohen’s d for the post-intervention comparison between TSG and control groups.

Similar trends were observed for ${\dot{\text{V}}\text{O}}_{2\max}$, where TSG demonstrated within-group improvements across studies as Hornstrup et al. [48], Seabra et al. [42], Soares et al. [46] and Vasconcellos et al. [44]. Between-group comparisons favored TSG in most studies as Hornstrup et al. [48], Soares et al. [46] and Vasconcellos et al. [44], emphasizing the positive influence of team sports on aerobic performance.

## DISCUSSION

In the analysis of team sports recreational interventions on overweight and obese populations, distinct patterns emerge across various metabolic health indicators, body composition and physical fitness. Body composition and anthropometric measures, such as BMI and body fat percentage, consistently show favorable outcomes (small-to-moderate effect sizes) in team sports groups (TSG) compared to control groups (CG). On the cardiovascular health, TSG demonstrate a positive impact on SBP, indicating significant improvements and differences with CG. However, the influence on DBP appears less consistent. TSG showcase a potential positive influence (small-tomoderate effect sizes) on lipid profiles, with total cholesterol and triglyceride levels favoring TSG in most studies, although the positive effects on glucose seems to be less consistent. Aerobic fitness, assessed through the field-based tests and ${\dot{\text{V}}\text{O}}_{2\max}$, consistently improves within TSG, underscoring the beneficial impact of team sports on overall fitness. These findings collectively emphasize the potential of team sports interventions in addressing various metabolic health, body composition and physical fitness parameters among overweight and obese populations.

### The impact of recreational team sports interventions on anthropometry and body composition

The evidence presented in the analysis of recreational team sports interventions for overweight and obese populations suggests a multifaceted positive impact on various anthropometric and body composition parameters. The consistent decrease in BMI and body fat percentage within TSG, as observed in studies by Cvetkovic et al. [25], Soares et al. [14] or Seabra et al. [12] underscores the effectiveness of team sports in addressing weight-related concerns. Although the results were not completely unanimous across the studies as the cases of Hornstrup et al. [48] or Soares et al. [46], it is possible to disclose a trend for TSG being favorable for enhancing critical body composition parameters.

It is important to note that the fight against fat mass and the enhancement of body mass composition cannot rely solely on physical efforts. For example, combining team sports with other strategies such as nutritional plans and education, as well as considering activity and lifestyle, is crucial. This is evident in studies that combined a dietary plan with TSG, compared to running-based high-intensity interval training or a control group employing only a dietary plan [46]. The results show enhancements in BMI and fat mass within the combined dietary plan and TSG group, although not significantly different from the control group [46].

The physiological benefits of TSG that commonly use small-sided games may lie in the high-intensity intermittent nature of the activities, promoting substantial caloric expenditure. Studies, including those by Toh et al [50], demonstrate that the metabolic demands of SSGs in invasion sports as soccer, basketball or handball contribute to increased involvement and energy expenditure. Furthermore, SSGs induce an elevated post-exercise lowered blood glucose concentration [51], as well as postprandial lipemia [52], leading to a more favorable adaptation in health parameters and ultimately body composition. Improved insulin sensitivity, a key factor in managing body weight, is also observed in small-sided games [53]. Additionally, the social and team-based aspects of SSGs, contribute to enhanced adherence, fostering a positive behavioral environment that promotes long-term engagement and, subsequently, significant improvements in fat mass and BMI among overweight and obese populations [54].

### The impact of recreational team sports interventions on metabolic health parameters

The comparison of TSG against CG across various studies provides valuable insights into the potential impact on metabolic health markers. Focusing on blood pressure, when examining SBP within the TSG, there were variations across studies, with some showing a significant decrease, such as the cases of Mohr et al. [49] and Vasconcellos et al. [44]. Between-group comparisons supported a significant favoring of TSG in terms of SBP in several studies as Hansen et al. [41], Mohr et al. [49], Moller et al. [47] and Vasconcellos et al. [44] suggesting a potential positive impact of team sports on SBP. SSG involves dynamic and intermittent physical activities, including rapid accelerations, decelerations, and changes in direction [55]. These characteristics contribute to increased cardiovascular demands and enhanced aerobic fitness [56]. Regular participation in SSG has been shown to improve endothelial function, reduce arterial stiffness, and enhance vasodilatory capacity [41]. The cumulative effect of these physiological adaptations likely leads to a reduction in systemic vascular resistance, ultimately contributing to the observed improvement in SBP. In contrast, the impact of TSG on DBP exhibited more variability, with only one study, Mohr et al. [49], indicating a significant decrease. The majority of betweengroup comparisons did not confirm a significant and positive impact of team sports on DBP, highlighting a need for further investigation in this regard.

Moving on to lipid profiles, the assessment of total cholesterol levels within the TSG showed mixed results, with some studies reporting a significant decrease as Seabra et al. [42], Soares et al. [46] and Vasconcellos et al. [44]. Moreover, between-group comparisons consistently favored TSG in terms of total cholesterol, suggesting a potential positive impact on this marker. Regular participation in TSG has been associated with increased high-density lipoprotein cholesterol, and decreased low-density lipoprotein cholesterol as demonstrated in Soares et al. [13].

Triglyceride levels within the TSG demonstrated a significant decrease across studies as Mohr et al. [49], Vasconcellos et al. [44] and Vasconcellos et al. [45]. The between-group comparisons further supported a significant favoring of TSG, underscoring a potential positive impact of team sports on triglyceride levels. The use of small-sided games with a more metabolic demands and high-intensity efforts may justify the stimulation of lipid oxidation, reducing triglyceride synthesis and storage [57].

Regarding glucose levels, within-group differences in TSG varied, with only one study Vasconcellos et al. [44] showing a significant decrease. While small-sided games used by TSG promotes cardiovascular fitness and contributes to weight management, its specific impact on glucose regulation may be influenced by individual metabolic differences, adherence levels, and the duration of the intervention. It is possible that the intermittent and varied nature of smallsided games may not provide a sustained stimulus for optimizing glucose metabolism compared to more continuous and structured exercise regimens [58].

### The impact of recreational team sports interventions on physical fitness

The analysis of studies comparing TSG against CG in terms of aerobic fitness, as measured by the field-based tests, reveals consistent and positive trends. Within-group improvements in TSG were evident in studies conducted by Hornstrup et al. [48], Mohr et al. [49] and Cvetkovic et al. [25], with significant increases in the distance covered. This pattern, consistently favoring TSG, indicates a substantial positive impact on aerobic fitness when compared to CG. Similar trends were observed for ${\dot{\text{V}}\text{O}}_{2\max}$ across various studies, including those by Hornstrup et al. [48], Seabra et al. [42], Soares et al. [46], and Vasconcellos et al. [44]. These findings collectively suggest that engagement in team sports leads to notable enhancements in aerobic fitness parameters compared to control groups, emphasizing the potential of team sports as an effective intervention for improving cardiovascular endurance. The dynamic and intermittent nature of small-sided games involving high metabolic demands can explain the improvement. The varied high intensity and duration of small-sided games stimulate adaptations in muscle mitochondria, increasing their efficiency in utilizing oxygen for energy production [59].

Moreover, the dynamic and high-intensity nature of TSG by using small-sided games necessitates increased oxygen delivery to active muscles, leading to heightened cardiac output. This heightened demand manifests as an elevated heart rate, a response indicative of the heart’s intensified effort to meet metabolic requirements [55]. With consistent exposure to such stimuli, the cardiovascular system undergoes adaptations, including improvements in stroke volume and cardiac output efficiency [60]. These adaptations contribute to heightened aerobic fitness by augmenting the heart’s ability to efficiently supply oxygenated blood to exercising muscles. The intricate interplay between intensified exercise, cardiovascular adaptations, and improved aerobic capacity underscores the scientific rationale behind the observed enhancement in aerobic fitness resulting from regular engagement in intense activities like small-sided games in TSG.

### Study limitations, future research and practical implications

While the findings of this analysis highlight the positive impact of recreational team sports interventions on various metabolic health indicators, body composition, and physical fitness in overweight and obese populations, certain limitations must be acknowledged. Firstly, the heterogeneity in study designs, intervention durations, and participant characteristics across the included studies may contribute to variations in the observed outcomes. Additionally, the reliance on self-reported dietary and lifestyle information in some studies introduces the potential for measurement bias and limits the precision of associations between team sports interventions and metabolic health improvements. Furthermore, the generalizability of the results may be influenced by the specific team sports included in the interventions and the unique characteristics of the study populations. These limitations underscore the need for future research to employ standardized methodologies and larger, more diverse samples to enhance the generalizability and robustness of the findings.

Future research endeavors should address the identified limitations by adopting more rigorous study designs, including randomized controlled trials with well-defined protocols for team sports interventions. Long-term follow-up assessments are crucial to elucidate the sustainability of the observed improvements in metabolic health markers and physical fitness. Moreover, investigating the optimal frequency, intensity, duration, and types of team sports activities for achieving specific health outcomes will provide valuable insights for designing targeted interventions. Additionally, exploring the potential moderating effects of individual characteristics, such as age, sex, and baseline fitness levels, will contribute to a nuanced understanding of the differential responses to team sports interventions.

The positive impact of recreational team sports interventions on metabolic health, body composition, and physical fitness observed in this analysis holds promising implications for practical applications in addressing overweight and obesity. Health practitioners, fitness professionals, and policymakers can consider incorporating team sports activities, especially those involving small-sided games, into lifestyle interventions for weight management and overall health improvement. In general, soccer training groups commonly incorporate three to four sessions per week, lasting 60 to 90 minutes each, using small-sided games formats from 4 v 4 to 8 v 8. Sessions typically include warm-ups, technical drills, small-sided games, and cool-downs.

The social and enjoyable nature of team sports may enhance adherence, making them an attractive and sustainable option for individuals with overweight and obesity. Moreover, combining team sports with tailored nutritional plans and educational components may amplify the synergistic effects on metabolic health. Implementing community-based team sports programs, particularly those that target specific health outcomes, can serve as a feasible and engaging strategy to combat the rising prevalence of overweight and obesity, promoting a holistic approach to health and well-being.

## CONCLUSIONS

In conclusion, the systematic review of team sports recreational interventions for overweight and obese populations reveals consistent and favorable outcomes across various metabolic health indicators, body composition, and physical fitness parameters. Team sports groups consistently exhibit positive effects on body composition, including a notable decrease in BMI and body fat percentage. The cardiovascular benefits of TSG are evident in the significant improvements in systolic blood pressure, although the impact on diastolic blood pressure appears less consistent. TSG also demonstrate potential positive influences on lipid profiles, with total cholesterol and triglyceride levels favoring TSG in most studies. Additionally, aerobic fitness consistently improves within TSG, emphasizing the overall positive impact of team sports on fitness. However, variations in glucose outcomes suggest a need for further investigation. Future investigations should focus on optimizing team sports interventions and exploring individual moderating factors. The practical implications highlight the potential for health practitioners and policymakers to integrate team sports, particularly those involving small-sided games, into lifestyle interventions for effective weight management and overall health improvement. In the future, it is expected that governments and health systems will integrate recreational team sports under the guidance of sports technical staff in community-based structured programs. This approach is seen as a key strategy to prevent non-communicable diseases and offer advantages to overweight and obese populations. It is an integral component of a multidisciplinary response to the challenges presented by changing lifestyles.

## Supplementary Material

Effects of recreational team sports on the metabolic health, body composition and physical fitness parameters of overweight and obese populations: A systematic review
