# Supplementary material for: Effects of recreational team sports on the metabolic health, body composition and physical fitness parameters of overweight and obese populations: A systematic review
Source: Biol Sport. 2024 Feb 12;41(3):243–66. doi: 10.5114/biolsport.2024.134762 (PMC11167458; doi:10.5114/biolsport.2024.134762)
Supplement: Effects of recreational team sports on the metabolic health, body composition and physical fitness parameters of overweight and obese populations: A systematic review [file JBS-41-52319-s1.pdf]

SUPPLEMENTARY FILE 1. Full-text screening.

STUDY	POPULATION	INTERVENTION	COMPARATOR	OUTCOME	DESIGN	DECISION
Calcaterra, V., Larizza, D., Codrons, E., De Silvestri, A., Brambilla, P., Abela, S., … Vandoni, M. (2013). Improved metabolic and cardiorespiratory fitness during a recreational training program in obese children. Journal of Pediatric Endocrinology and Metabolism, 26(3–4), 271–276. doi:10.1515/jpem-2012-0157	Y	Y	N	Y	N	Excluded
Gray, C. M., Hunt, K., Mutrie, N., Anderson, A. S., Leishman, J., Dalgarno, L., & Wyke, S. (2013). Football Fans in Training: The development and optimization of an intervention delivered through professional sports clubs to help men lose weight, become more active and adopt healthier eating habits. BMC Public Health, 13(1). doi:10.1186/1471-2458-13-232	Y	N	N	N	N	Excluded
Gray, C. M., Hunt, K., Mutrie, N., Anderson, A. S., Treweek, S., & Wyke, S. (2013). Weight management for overweight and obese men delivered through professional football clubs: A pilot randomized trial. INTERNATIONAL JOURNAL OF BEHAVIORAL NUTRITION AND PHYSICAL ACTIVITY, 10. doi:10.1186/1479-5868-10-121	Y	N	Y	Y	Y	Excluded
Bendiksen, M., Williams, C. A., Hornstrup, T., Clausen, H., Kloppenborg, J., Shumikhin, D., … Krustrup, P. (2014). Heart rate response and fitness effects of various types of physical education for 8- to 9-year-old schoolchildren. EUROPEAN JOURNAL OF SPORT SCIENCE, 14(8), 861–869. doi:10.1080/17461391.2014.884168	N	Y	Y	Y	Y	Excluded
Mohr, M., Lindenskov, A., Holm, P. M., Nielsen, H. P., Mortensen, J., Weihe, P., & Krustrup, P. (2014). Football training improves cardiovascular health profile in sedentary, premenopausal hypertensive women. Scandinavian Journal of Medicine and Science in Sports, 24(SUPPL.1), 36–42. doi:10.1111/sms.12278	Y	Y	Y	Y	Y	INCLUDED
Rutherford, Z., Gough, B., Seymour-Smith, S., Matthews, C. R., Wilcox, J., Parnell, D., & Pringle, A. (2014). 'Motivate': the effect of a Football in the Community delivered weight loss programme on over 35-year old men and women's cardiovascular risk factors. Soccer and Society, 15(6), 951–969. doi:10.1080/14660970.2014.920628	Y	N	N	N	N	Excluded
Milanovic, Z., Pantelic, S., Kostic, R., Trajkovic, N., & Sporis, G. (2015). Soccer vs. running training effects in young adult men: which programme is more effective in improvement of body composition? Randomized controlled trial. BIOLOGY OF SPORT, 32(4), 301–305. doi:10.5604/20831862.1163693	N	Y	Y	Y	Y	Excluded
Wyke, S., Hunt, K., Gray, C. M., Fenwick, E., Bunn, C., Donnan, P. T., … Treweek, S. (2015). Football Fans in Training (FFIT): a randomised controlled trial of a gender-sensitised weight loss and healthy living programme for men – end of study report. Southampton (UK): NIHR Journals Library.	Y	N	Y	Y	Y	Excluded
Eichner, J. E., Folorunso, O. A., & Moore, W. E. (2016). A physical activity intervention and changes in body mass index at a middle school with a large American Indian Population, Oklahoma, 2004–2009. Preventing Chronic Disease, 13(12). doi:10.5888/pcd13.150495	N	N	N	N	N	Excluded

SUPPLEMENTARY FILE 1. Continue

STUDY	POPULATION	INTERVENTION	COMPARATOR	OUTCOME	DESIGN	DECISION
Gill, D. P., Blunt, W., De Cruz, A., Riggin, B., Hunt, K., Zou, G., … Petrella, R. J. (2016). Hockey Fans in Training (Hockey FIT) pilot study protocol: A gender-sensitized weight loss and healthy lifestyle program for overweight and obese male hockey fans. BMC Public Health, 16(1). doi:10.1186/s12889-016-3730-5	Y	N	N	N	N	Excluded
Seabra, A., Katzmarzyk, P., Carvalho, M. J., Coelho-E-Silva, M., Abreu, S., Vale, S., … Malina, R. M. (2016). Effects of 6-month soccer and traditional physical activity programmes on body composition, cardiometabolic risk factors, inflammatory, oxidative stress markers and cardiorespiratory fitness in obese boys. JOURNAL OF SPORTS SCIENCES, 34(19), 1822–1829. doi:10.1080/02640414.2016.1140219	Y	Y	Y	Y	Y	INCLUDED
Vasconcellos, F., Seabra, A., Cunha, F., Montenegro, R., Penha, J., Bouskela, E., … Farinatti, P. (2016). Health markers in obese adolescents improved by a 12-week recreational soccer program: a randomised controlled trial. JOURNAL OF SPORTS SCIENCES, 34(6), 564–575. doi:10.1080/02640414.2015.1064150	Y	Y	Y	Y	Y	INCLUDED
Azman, N., Mohamed, N. G., Aziz, A. R., Farah, N., & Muhamed, A. M. C. (2018). The effectiveness of futsal as a game-simulated exercise for promoting weight loss and metabolic health in overweight/obese men. Malaysian Journal of Public Health Medicine, 2018(Specialissue1), 174–182. Retrieved from https://www.scopus.com/inward/record.uri?eid=2-s2.0-85044829530&partner ID=40&md5=ae948d8cee94c8b09417f178696bf689	Y	Y	N	Y	N	Excluded
Cvetković, N., Stojanović, E., Stojiljković, N., Nikolić, D., Scanlan, A. T., & Milanović, Z. (2018). Exercise training in overweight and obese children: Recreational football and high-intensity interval training provide similar benefits to physical fitness. Scand J Med Sci Sports, 28 Suppl 1, 18–32. doi:10.1111/sms.13241	Y	Y	Y	Y	Y	INCLUDED
Gray, C. M., Wyke, S., Zhang, R. Q., Anderson, A. S., Barry, S., Boyer, N., … Hunt, K. (2018). Long-term weight loss trajectories following participation in a randomised controlled trial of a weight management programme for men delivered through professional football clubs: a longitudinal cohort study and economic evaluation. INTERNATIONAL JOURNAL OF BEHAVIORAL NUTRITION AND PHYSICAL ACTIVITY, 15. doi:10.1186/s12966-018-0683-3	Y	N	Y	Y	N	Excluded
Jaafar, Z., Kee, J., Abdul Hadi, H., & Ahmad Tajuddin, N. A. (2018). Anthropometrical and fitness level changes following a 12-week walking football program for obese primary school children aged 8–11. Medicina dello Sport, 71(3), 451–460. doi:10.23736/s0025-7826.18.03285-4	Y	Y	N	Y	N	Excluded
Møller, T. K., Nielsen, T. T., Andersen, R., Lundager, I., Hansen, H. F., Ottesen, L., … Randers, M. B. (2018). Health Effects of 12 Weeks of Team-Sport Training and Fitness Training in a Community Health Centre for Sedentary Men with Lifestyle Diseases. BioMed Research International, 2018. doi:10.1155/2018/1571807	Y	Y	Y	Y	Y	INCLUDED

SUPPLEMENTARY FILE 1. Continue

STUDY	POPULATION	INTERVENTION	COMPARATOR	OUTCOME	DESIGN	DECISION
Frediani, J. K., Bienvenida, A. F., Li, J., Higgins, M. K., & Lobelo, F. (2020). Physical fitness and activity changes after a 24-week soccer-based adaptation of the U.S diabetes prevention program intervention in Hispanic men. Progress in Cardiovascular Diseases, 63(6), 775–785. doi:10.1016/j.pcad.2020.06.012	Y	Y	N	Y	N	Excluded
Hornstrup, T., Póvoas, S., Helge, J. W., Melcher, P. S., Fristrup, B., Andersen, J. L., … Krustrup, P. (2020). Cardiovascular and metabolic health effects of team handball training in overweight women: Impact of prior experience. SCANDINAVIAN JOURNAL OF MEDICINE & SCIENCE IN SPORTS, 30(2), 281–294. doi:10.1111/sms.13563	Y	Y	Y	Y	Y	INCLUDED
Mielke, G. I., Bailey, T. G., Burton, N. W., & Brown, W. J. (2020). Participation in sports/recreational activities and incidence of hypertension, diabetes, and obesity in adults. Scandinavian Journal of Medicine and Science in Sports, 30(12), 2390–2398. doi:10.1111/sms.13795	Y	N	N	Y	N	Excluded
Broglio, L. P., Gonelli, P. R. G., Costa, C. O., Sajorato, T. C., Massarutto, V., & Cesar, M. C. (2021). Volleyball as an exercise program for overweight and obese female adolescents. Revista Brasileira de Medicina do Esporte, 27(6), 545–548. doi:10.1590/1517-869220212706169680	N	Y	N	Y	N	Excluded
Frediani, J. K., Li, J., Bienvenida, A., Higgins, M. K., & Lobelo, F. (2021). Metabolic Changes After a 24-Week Soccer-Based Adaptation of the Diabetes Prevention Program in Hispanic Males: A One-Arm Pilot Clinical Trial. Frontiers in Sports and Active Living, 3. doi:10.3389/fspor.2021.757815	Y	Y	N	Y	N	Excluded
Vasconcellos, F., Cunha, F. A., Gonet, D. T., & Farinatti, P. T. V. (2021). Does Recreational Soccer Change Metabolic Syndrome Status in Obese Adolescents? A Pilot Study. Research Quarterly for Exercise and Sport, 92(1), 91–99. doi:10.1080/02701367.2019.1711007	Y	Y	Y	Y	Y	INCLUDED
Colpitts, B. H., Keshavarz, M., Blake, M., Sénéchal, M., Gallibois, M., Olthuis, J., … Bouchard, D. R. (2022). Health benefits associated with the Hockey Fans in Training® program for overweight men who are university hockey fans. SCIENCE & SPORTS, 37(1). doi:10.1016/j.scispo.2020.12.009	Y	Y	N	Y	N	Excluded
da Silva Soares, D. B., Shinjo, S. K., Santos, A. S., de Cassia Rosa de Jesus, J., Schenk, S., de Castro, G. S., … de Sousa, M. V. (2022). Skeletal muscle gene expression in older adults with type 2 diabetes mellitus undergoing calorie-restricted diet and recreational sports training – a randomized clinical trial. EXPERIMENTAL GERONTOLOGY, 164. doi:10.1016/j.exger.2022.111831	Y	Y	Y	Y	Y	INCLUDED

SUPPLEMENTARY FILE 1. Continue

STUDY	POPULATION	INTERVENTION	COMPARATOR	OUTCOME	DESIGN	DECISION
Maddison, R., Hargreaves, E. A., Jiang, Y., Calder, A. J., Wyke, S., Gray, C. M., … Marsh, S. (2023). Rugby Fans in Training New Zealand (RUFIT NZ): a randomized controlled trial to assess the effectiveness of a healthy lifestyle program for overweight men delivered through professional rugby clubs. INTERNATIONAL JOURNAL OF BEHAVIORAL NUTRITION AND PHYSICAL ACTIVITY, 20(1). doi:10.1186/s12966-022-01395-w	Y	N	Y	Y	Y	Excluded
Pinho, C. D. F., Farinha, J. B., Lisboa, S. D. C., Bagatini, N. C., Leites, G. T., Voser, R. D. C., … Cunha, G. S. (2023). EFFECTS OF A SMALL-SIDED SOCCER PROGRAM ON HEALTH PARAMETERS IN OBESE CHILDREN. Revista Brasileira de Medicina do Esporte, 29. doi:10.1590/1517-8692202329012021_0398	Y	Y	N	Y	N	Excluded
Soares, I. F., Cunha, F. A., & Vasconcellos, F. (2023). Effects of a 12-Week Recreational Soccer Program on Resting Metabolic Rate Among Adolescents with Obesity. Journal of Science in Sport and Exercise, 5(3), 218–225. doi:10.1007/s42978-022-00181-1	Y	Y	Y	Y	Y	INCLUDED

EXTRA						
Hansen, P. R., Andersen, L. J., Rebelo, A. N., Brito, J., Hornstrup, T., Schmidt, J. F., … & Krustrup, P. (2013). Cardiovascular effects of 3 months of football training in overweight children examined by comprehensive echocardiography: a pilot study. Journal of sports sciences, 31(13), 1432–1440.	Y	Y	Y	Y	Y	Included
